# The With Or Without Olecranon K-wire (WOW OK) Trial of tension band wire fixation versus cerclage fixation without K-wires in displaced stable olecranon fractures: study protocol for a randomized controlled trial

**DOI:** 10.1186/s13063-023-07566-9

**Published:** 2023-08-29

**Authors:** Gustav Cornefjord, Ioannis Kostogiannis, Cecilia Rogmark, Daniel Jerrhag, Daniel Wenger

**Affiliations:** 1 Department of Clinical Sciences Malmö, Clinical and Molecular Osteoporosis Research Unit, Lund University, Lund, Sweden; 2 Department of Orthopaedics, Skåne University Hospital, Malmö, Sweden

**Keywords:** Olecranon fractures, Fractures, Bone, Ulna fractures, Wounds and injuries, Forearm injuries, Arm injuries

## Abstract

**Background:**

Displaced olecranon fractures with a stable elbow joint are classified as Mayo type 2a or 2b and are commonly operated with tension band wiring, i.e. two K-wires and a cerclage. Retrospective studies have reported fewer reoperations and complications with cerclage fixation alone when compared to tension band wiring, though with similar long-term results. We decided to compare tension band wiring to cerclage fixation of displaced, stable olecranon fractures in adults in a randomized controlled trial.

**Methods:**

All patients ≥ 18 years old with Mayo type 2a and 2b fractures presenting at Skåne University hospital will be eligible for study inclusion, unless exclusion criteria are met. Two hundred participants will be included and randomized 1:1 to cerclage fixation or tension band wiring.

Outpatient physiotherapist follow-up appointments will be scheduled at 2 and 6 weeks and at 3, 12, and 36 months at the Dept. of Orthopaedics. A lateral view radiograph of the elbow will be analysed at 6 months. The primary outcome of our study is the rate of reoperations. Secondary outcomes are complication rates, severity of complications, and patient-reported outcome measures (*Quick*DASH, Short Musculoskeletal Function Assessment, pain level, and patient satisfaction). The sample size was calculated to give 80% power for detecting a statistically significant difference in reoperation rates (with alpha-value 0.05), based on a previous retrospective study.

**Discussion:**

Reoperation and complication rates after tension band wiring of olecranon fractures are high. Treatment of these injuries is debated, and several ongoing trials compare tension band wiring with plate fixation, suture fixation, and non-operative treatment. As data from retrospective studies indicate that cerclage fixation may be superior to tension band wiring, we see a need for a randomized controlled trial comparing these methods. The WOW-OK Trial aims to obtain level-1 evidence that may influence treatment choice for this type of fracture.

**Trial registration:**

ClinicalTrials.gov NCT05657899. Registered on 16 November 2022. The trial complies with SPIRIT and CONSORT guidelines. The SPIRIT figure is found in Table 2.

**Supplementary Information:**

The online version contains supplementary material available at 10.1186/s13063-023-07566-9.

## Introduction

Olecranon fractures account for approximately 20% of all fractures of the proximal forearm [1]. Stable, undisplaced fractures are routinely treated non-operatively [2–4], while displaced fractures with instability in the ulno-humeral joint (so called trans-olecranon dislocations) are commonly operated with plate fixation [2, 5]. Displaced fractures that leave the elbow joint stable (Mayo type 2a and 2b) are often treated with tension band wiring (TBW) or plate fixation [2]. TBW is associated with soft tissue irritation, and high reoperation rates with hardware removal range from 25 to 84% in the literature [5–9]. Plate fixation has been associated with fewer reoperations but more severe complications compared to TBW in 2a and 2b fractures [10]. A Cochrane review from 2014 did not find any good support favouring either TBW or plate fixation over the other [11]. Nonoperative treatment of Mayo type 2a and 2b fractures yields acceptable outcomes in elderly patients with low functional demands but is not recommended to patients with moderate or high functional demands [12–15].

Previous retrospective studies at our department have shown that Mayo 2a and 2b fractures can be operated with cerclage fixation without K-wires (CF) yielding half the reoperation rate compared with TBW [7, 16]. To our knowledge, no other clinical studies have compared CF with TBW. As of 15 November 2022, there are 6 studies comparing treatments for olecranon registered on ClinicalTrials.gov. These included the following: one active and one completed study comparing TBW with plate fixation [17, 18], two active and one terminated study comparing surgical with non-surgical treatment [19–21], and one active study comparing TBW with suture fixation are registered [22]. Also, one active study comparing TBW with suture fixation is found in the International Standard Randomized Controlled Trial Number (ISRCTN) registry [23].

The controversy over treatment method for Mayo 2a and 2b fractures elicits the need to examine our research question: Is outcome following CF better than TBW for Mayo type 2a and 2b olecranon fractures? Our hypotheses are that CF will result in lower reoperation rate, complication rate, and intra-operative radiation time and exposure compared with TBW, while yielding non-inferior results in our specified secondary and tertiary outcome measures, including the patient related outcome *Quick*DASH. We aim to investigate this in a prospective 1:1 randomized controlled trial (RCT).

## Methods

### Trial summary

All patients presenting at Skåne University Hospital in Malmö or Lund with olecranon fractures of Mayo type 2a and 2b will be eligible for inclusion in the trial. Participants meeting exclusion criteria will be excluded. Informed voluntary written consent will be required for participation. Participants will be randomized 1:1 to surgery with CF or TBW.

Baseline descriptive data will be collected, and participants will be invited to a clinical follow-up at a physiotherapist outpatient clinic at the Dept. of Orthopaedics for collection of outcome measures at 2 and 6 weeks and 3, 12 and 36 months after surgery. The physiotherapist will be blinded to the treatment method of the participants. At 6 months, a lateral elbow radiograph will be performed and assessed for secondary dislocation and/or nonunion by an orthopaedic consultant.

The primary outcome measure is reoperation rates. Secondary outcome measures are complication rates and patient-reported outcome measures (PROM). Several tertiary outcome measures will be collected. Our hypothesis is that reoperation rates, complication rates, and intra-operative radiation time and exposure will be lower in the CF group while other outcome measures will be non-inferior for CF.

### Null hypothesis

The null hypothesis in this study is that there is no difference in outcome measures at the follow-up time-points in adult participant with Mayo type 2a and 2b olecranon fractures treated with CF and TBW. Our alternative hypothesis is that CF yields superior results with fewer reoperations, fewer overall and severe complications, lower intra-operative radiation time and exposure, and non-inferior results in other outcomes.

### Objectives and outcome measures

The primary outcome measure is reoperation rates. A reoperation is cumbersome and costly and exposes the patient to additional risks associated with further surgical and anaesthesiologic procedures. Because of its significance for both patients and health-care systems, and as previous data indicate a large difference in reoperations between the two methods, reoperation rates was chosen as the primary endpoint.

The secondary outcome measures are:

- Complication rates based on a complication defined as any negative unexpected event attributed to the treatment.
- Severe complication rate is a constructed variable. Complications will be classified by the study official according to the modified Clavien-Dindo classification, a score specific for complications to orthopaedic surgery [24, 25]. Any complication of level 3a, 3b or higher is considered severe, thus creating a dichotomous variable (severe complication/not severe complication).
- The Quick Disabilities of the Arm, Shoulder and Hand score (*Quick*DASH) is a self-assessed questionnaire consisting of 11 items graded 1–5 assessing upper limb function for the affected arm, resulting in a 0–100 score [26]. It was chosen as it is a validated instrument [27], which is widely used throughout the literature, enhancing comparability of the results. The *Quick*DASH questionnaire performs similar to its full-length version the Disabilities of the Arm, Shoulder and Hand score [28].
- The Short Musculoskeletal Function Assessment (SMFA) questionnaire is a validated 46-item questionnaire used to assess overall musculoskeletal function [27, 29]. The result is converted to a 0–100 score with perceived function decreasing with increasing score. The interpretation of SMFA depends on translation and cultural adaptation. The Swedish adaptation is used in this trial, using bother (bSMFA) and dysfunction (dSMFA) indices as suggested by Williams [29].
- Overall satisfaction will be self-reported using a visual analogue scale (VAS) from 0 to 100. Zero corresponds to “completely satisfied” and 100 to “not satisfied at all”.
- Pain in rest and motion will be self-reported with VAS from 0 to 100. Zero corresponds to “no pain at all” and 100 to “worst imaginable pain” in the elbow.

The tertiary outcome measures are:

- Grip strength will be evaluated using a JAMAR dynamometer according to the guidelines from the Swedish national quality register for hand surgery (HAKIR) [30]. The dynamometers used will be calibrated at least once every year.
- Active postoperative range of motion (ROM) for extension, flexion, pronation and supination will be evaluated using the HAKIR guidelines [30].
- Secondary dislocation and/or nonunion rate will be assessed with a lateral view radiograph 6 months after the operation. In order to minimize radiation dose, only one radiograph and one view will be obtained. If secondary dislocation and/or nonunion is found, the participant will be referred to the standard healthcare system, and further investigation may include a full radiographic examination. Any demonstrated secondary dislocation and/or nonunion is regarded as a complication.
- Sick leave related to the elbow injury, counted in days.
- Intra-operative fluoroscopy radiation level will be measured as the dose area product (DAP) in mGy·cm^2^, and intraoperative fluoroscopy time will be measured in seconds. This will be recorded at the time of surgery from the fluoroscopy machine used. The model and make of the machine vary between operating rooms and may change during the study.
- Surgical time will be recorded in minutes during surgery counting from skin incision to the final suture of the surgical wound.
- Post-operative antibiotic treatment is defined as any prescribed antibiotic treatment related to the surgery after the intervention.

### Sample characteristics

The study will be conducted at Skåne University Hospital serving the towns of Malmö and Lund in southern Sweden. The hospital is a tertiary centre for orthopaedic trauma and an academic centre for orthopaedic research. The hospital serves a defined geographic catchment area, with no other hospital that treats olecranon fractures in that area. Patients referred with olecranon fractures from other hospitals, for example due to severe soft-tissue injury or multi-trauma, will not be eligible for inclusion. To further characterize the study sample, descriptive statistics on age, gender, dominant hand, Mayo subclass (2a or 2b), high energy trauma, and current smoking (during the last month) will be recorded at inclusion or in the peri-operative period.

### Sample size

The primary outcome measure of the study is reoperation rates, and the sample size is based on this metric. Reoperation rates are assumed to be 13% for CF and 27% for TBW, based on a previous study at our institution [16]. As active patient participation is not required to calculate reoperation rates, and as it will be collected in the same health-care system as the previous retrospective study, we do not expect any drop-out of participants (relative to that study) for assessment of reoperations. The sample size of each arm needed to achieve 80% power is 98; thus, we plan to recruit a total of 200 participants, yielding 81% power.

We expect all 200 participants to be available for analyses of peri-operatively recorded variables (age, sex, fracture type, fluoroscopy DAP, fluoroscopy time and operation time). For analyses of complications, the same assumption is made as for reoperations, i.e. no drop-out relative to our previous retrospective study. For all other variables collected at follow-up, we expect 170 participants to be available for statistical analysis. The expected power for all variables at different sample sizes are shown in Table 1, including all relevant assumptions made.

**Table 1:** Statistical power and sample size requirements for study outcomes

	**Power *n* = 200**	**Power *n* = 170**	***n* needed for 80% power**	**MDD *n* = 100**	**MDD**	**MDD *n* = 170**	**MDD**	**x̄ or proportion**	**SD**	**MCID**
**Primary measure**										
Reoperation rate (yes/no)	81%	75%	196	0.2	0.16	0.15	0.14	13% vs 27%^a^	-	-
**Secondary measures**										
Complication rate (yes/no)	> 99%	99%	68	0.22	0.18	0.16	0.15	18% vs 45%^a^	-	-
Severe complication rate (yes/no)	81%	75%	196	0.2	0.16	0.15	0.14	13% vs 27%^a^	-	-
* Quick*DASH (score)	94%	89%	138	12.9	10.5	9.9	9.1	13^c^	22^d^	14^e^
Patient satisfaction (VAS)	> 99%	> 99%	36	1.2	1	0.9	0.8	5^b^	2^b^	2^b^
Pain at rest (VAS)	94%	89%	138	1.2	1	0.9	0.8	1^b^	2^b^	1^b^
Pain in motion (VAS)	94%	89%	138	1.2	1	0.9	0.8	2^b^	2^b^	1^b^
bSMFA (score)	94%	89%	138	10.5	8.6	8.2	7.4	27^f^	18^f^	9^g^
dSMFA (score)	94%	89%	138	10.5	8.6	8.2	7.4	30^f^	18^f^	9^g^
**Tertiary measures**										
ROM, flexion (degrees)	> 99%	> 99%	18	4.1	3.3	3.1	2.9	146^h^	7^h^	10^b^
ROM, extension (degrees)	> 99%	> 99%	10	2.9	2.4	2.2	2.1	− 3^h^	5^h^	10^b^
ROM, supination (degrees)	> 99%	> 99%	14	3.5	2.9	2.7	2.5	80^h^	6^h^	10^b^
ROM, pronation (degrees)	> 99%	> 99%	18	4.1	3.3	3.1	2.9	87^h^	7^h^	10^b^
Grip strength (newton)	43%	31%	352	9.4	7.6	7.2	6.6	32^i^	16^i^	5 ^j^
Post-operative antibiotics (yes/no)	< 1%	< 1%	6296	16%	13%	12%	11%	8%^a^	-	2%^b^
Secondary nonunion (yes/no)	< 1%	< 1%	9782	< 0.1%	< 0.1%	< 0.1%	< 0.1%	1%^k^	-	0.5%^b^
Sick leave (days)	< 1%^n^	< 1%^l^	30,790	18	14	13	12	35^b^	302	12
Fluoroscopy time (seconds)	< 1%	< 1%	3024	55	45	42	39	81^m^	94^m^	10^b^
Fluoroscopy DAP (mGy·cm^2^)	< 1%	< 1%	1492	19	16	15	14	25^m^	33^m^	5^b^
Operation time (minutes)	< 1%	< 1%	998	15	13	12	11	83^m^	27^m^	5^b^

Calculations of power and sample size requirements (n) for all outcome measures, including underlying assumptions. Previously published Minimally Clinically Important Differences (MCID) are given for outcomes where non-inferiority testing is performed and are used as non-inferiority margins in statistical analyses

^a^Based on a previous study at our institution [16]

^b^Based on estimations by the authors

^c^Based on QuickDASH following TBW [31]

^d^Based on unpublished data from a previous study where standard deviation (SD) was 22 for CF and TBW, although the sample was small

^e^Ranging from 3.5 to 19 points in the literature with a mean of 14 points [32–36]

^f^Based on outcomes following distal radius fractures as no elbow specific data was found in the literature [37]

^g^To our knowledge, no studies have reported MCID of SMFA following surgery for upper extremity injuries. The MCID was estimated to be half the standard deviation [38]

^h^Based on normal ROM [39]

^i^Based on data on elbow fractures [40]

^j^Based on a review of MCID of grip strength [41]

^k^Based on a study on the outcome of 196 olecranon fractures [42]

^l^Power if *n* = 100 and *n* = 85 as we expect half of the participant to work

^m^Based on data from all CF and TBW cases at our department during 2020

### Eligibility and exclusion

All patients presenting at Skåne University Hospital > 17 years old with a Mayo type 2a or 2b olecranon fracture will be eligible for the study. Patients living in our catchment area but who sustain an olecranon fracture outside the area will be eligible for inclusion if treated at our hospital. The exclusion criteria are:

- Participants unable to participate in follow-up (for example due to active substance use disorder, dementia, inability to communicate or understand the questionnaires (for example not speaking Swedish), or living in other administrative health-care region).
- Participants unable or unwilling to give informed written consent.
- Participants where non-operative treatment is indicated (for example frail patients).
- Participants with severe open fractures of Gustilo-Anderson type III.
- Participants with pathological fractures.
- Participants that are treated with surgery later than 14 days from the initial trauma.
- Participants operated by a surgeon who has not attended education on the present study and surgical methods provided by the study officials. If surgery is supervised by a surgeon that has attended the education, the participant will not be excluded.

If a participant meets any of the exclusion criteria, the study officials will exclude them from the study. An anonymous record of the number of eligible but excluded participant, including reason for exclusion, will be kept by the study officials.

### Enrolment and consent

Eligible participants will be identified at the emergency department by the initially treating physician or by any treating physician or steering committee at any time point prior to intervention. Upon identification, potential participants will receive consent forms, written and oral information about the study from the steering committee. Participation is voluntary and participants will be required to sign a written consent form. Participants who decline participation will receive treatment according to the treating physicians’ preferences. The exclusion criteria include inability to understand the given information or written consent. Thus, no consent will be presumed. Recruitment will end when 200 participants are included.

### Allocation and randomization

Prior to commencement of the study, a randomization sequence will be created by a research assistant using a tool provided by the US National Cancer Institute (NCI) [43]. The sequence will be created using maximally tolerated-imbalance (MTI) randomization and entered into the REDCap software [44, 45]. No one will be able to access the sequence once entered to into REDCap. Randomization will be stratified by age (≥ 65 years/ < 65 years), gender (male/female), and smoking (yes/no). These parameters are chosen as they are hypothesized to affect outcome and are available at inclusion.

Before the intervention, stratification data will be collected and the participant will be allocated to either intervention in the surgical ward just prior to when the patient enters the operating theatre by the steering committee using the sequence in the REDCap software, whereby a unique ID will be created for each participant.

### Blinding

Study participants will not be actively informed about which method was used but will be informed upon request. The physiotherapists measuring outcomes during follow-up will not be blinded to the treatment method; as they have access to the hospital files, a reliable blinding will be impossible to achieve in a cost-effective way. The physician assessing secondary dislocation or nonunion cannot be blinded as the treatment method will become apparent in the radiograph analysis. The study officials, surgeons, and other health-care providers will not be blinded. Data analysis will be performed on a blinded data set.

### Intervention

Intervention is internal fixation by either CF or TBW in the corresponding study arms. All orthopaedic surgeons subspecialized in trauma at our department are familiar with both treatment methods and have attended an education organized by the study officials. Surgery may be performed by other surgeons if supervised by a surgeon who has attended the education. During the trial, no specific concomitant treatment is prohibited or controlled by the trial.

#### Intra-operative standards

All participants will receive prophylactic intravenous antibiotics as a single dose of 2 g cloxacillin (or 600 mg clindamycin if allergic to cloxacillin) 30 min prior to the surgery. Tourniquet, fracture forceps, provisional K-wires, and intra-operative fluoroscopy may be used at the surgeons' discretion. A straight, or slightly curved, dorsal approach to the olecranon will be used. Following fracture reduction and fixation, the stability of the fixation will be checked under fluoroscopy during elbow extension and flexion. In case of comminution of the proximal fragment, a supplementary tendon suture may be used to decrease the risk of secondary displacement with implant cut-out. The wound will be closed in layers, and dressings and an elbow cast splint will be applied.

#### Cerclage fixation

**Fig. 1:**
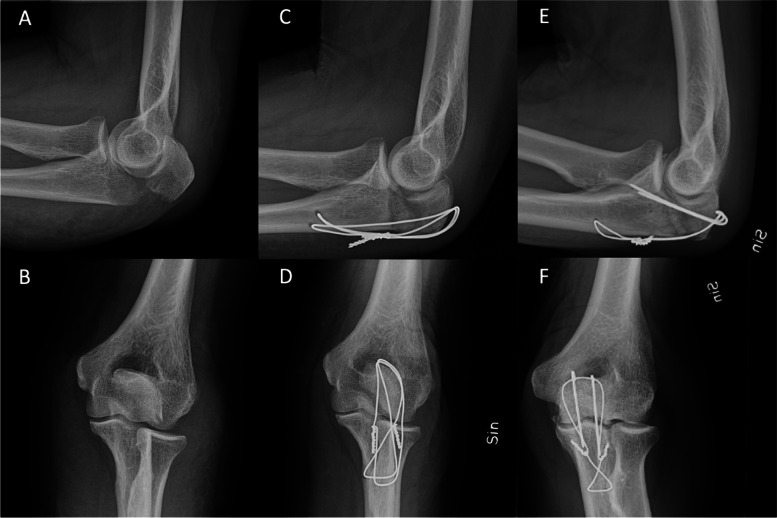
Fluoroscopy views of olecranon fractures. **A**, **B** Radiographs of olecranon type 2a fractures with a lateral (**A**) and an anterior–posterior view (**B**). Figure 1C and 1D shows radiographs following CF with a lateral (**C**) and an anterior–posterior view (**D**). **E**, **F** Radiographs following TBW with a lateral (**E**) and an anteriorposterior view (**F**)

The distal fragment will be drilled transversely using a 2.0-mm drill approximately 4 cm distally to the fracture line and 5 mm anterior to the posterior cortex. A cerclage will be put through the triceps tendon, just proximal to its attachment on the olecranon, passed over the fracture in a figure of eight configuration and through the drilled hole, and tightened. A second hole will be drilled at least 10 mm distal to the first hole and a cerclage will be placed through the second hole and the triceps attachment in a figure of zero figuration. Fluoroscopic images of CF are shown in Fig. 1.

#### Tension band wiring

The standard TBW method described in the AO guidelines will be used [46]. Fluoroscopic images of CF are shown in Fig. 1.

### Follow-up

After surgery, follow-up will be identical in both study groups (Table 2). Standard peri-operative care will be given at the hospital.

**Table 2:** Time-points for follow-up and data gathering, SPIRIT-figure

Activity	− 0.5 to 0	0	0.5	1.5	3	6	12	36
Inclusion, allocation, and randomization	x							
Intervention, both arms	x							
Primary outcome measures								
Reoperation rate				x	x		x	x
Secondary outcome measures								
Complication rate				x	x		x	x
Severe complication rate				x	x		x	x
QuickDASH				x	x		x	x
Patient satisfaction				x	x		x	x
Pain at rest				x	x		x	x
Pain in motion				x	x		x	x
SMFA		x					x	
Tertiary outcome measures								
ROM, flexion			x	x	x		x	x
Grip strength			x	x	x		x	x
Post-operative antibiotics				x	x		x	x
Secondary non union^a^			(x)	(x)	(x)	x	(x)	(x)
Sick leave				x	x		x	x
Fluoroscopy time		x						
Fluoroscopy DAP		x						
Operation time		x						

^a^The secondary dislocation or nonunion rate will be assessed from a lateral elbow radiograph at 6 months or will be recorded if diagnosed at any other time-point

In addition to descriptive baseline data, operation time, fluoroscopy DAP, and fluoroscopy time will be recorded. SMFA-questionnaires will be administered and collected by The Swedish Fracture Register (SFR) by mail shortly after the trauma (recall of pre-injury status), and 12 months later, and this data will be retrieved from SFR by the study officials.

At 2 weeks post-operatively, the cast will be removed, and a physiotherapist will record grip strength and ROM. Any reoperations, complications, ROM, grip strength, and return to work will be recorded at 6 weeks, 3 months, 12 months, and 36 months at the outpatient clinic at the dept. of Orthopaedics by a physiotherapist. PROMs (except SMFA) will be recorded by the participants in REDCap at the same time points. Reoperations and complications are recorded in each patient’s medical journal and subsequently retrieved by the study officials at interim analysis points and at the end of the trial.

At 6 months, a lateral view radiograph will be assessed by researchers DW and GC for any secondary dislocation or non-union. If secondary dislocation or nonunion is confirmed at any other point during the study, it will be recorded.

Any issue requiring additional follow-up or treatment will be handled in the standard health-care system which is public and tax-financed. After 36 months, the current follow-up will end.

### Post-inclusion withdrawals and exclusions

Participants included during the initial assessment may be excluded at any point post-inclusion if exclusion criteria are met. If consent has been given at this point, the data already recorded will be analysed. Participants may decide to voluntarily withdraw at any point, receiving standard healthcare. Upon withdrawal, participants may choose to allow previously and future health-care data to be analysed, only allow previously collected data to be analysed, or decline analysis of any such data.

### Adverse event management

Any adverse events will be treated in the standard health-care system. In such cases, there will be no difference to the standard care. All participants are fully insured and entitled to compensation through the Swedish Patient Injury Act, under the same conditions as patients not participating in the study.

### End of trial and early termination

Recruitment will end when 200 participants have been recruited.

Interim analyses will be performed by the data monitoring committee every 6 months, starting in May 2024. All participants that have completed the follow up at 6 weeks be included in the interim analyses. For ethical reasons, the recruitment will be terminated by the steering committee if any of the following criteria is met:

- A statistically significant difference of ≥ 10% in reoperation rate between the study groups
- A statistically significant difference of ≥ 10% in severe complications rate between the study groups
- A statistically significant difference of ≥ 20 *Quick*DASH points between the groups at any follow-up time-point

In case of termination of the study, participants that have already been recruited will complete follow-up. The study will end when all recruited participants have completed the follow-up, are excluded, or have otherwise withdrawn from the study.

### Statistical analysis

For power and sample size calculations, the alpha value was set to 0.05. Our hypothesis is that CF will yield lower reoperation and complication rates, lower intra-operative radiation, and less intra-operative fluoroscopy time; thus, one-sided tests of proportions were used for sample size requirements calculations [47]. For all other variables, tests for non-inferiority were used with assumed means for ratio scalar and interval variables and assumed proportions for nominal variables [47].

Statistical power and sample size requirements for demonstrating clinically significant differences between the groups were calculated using minimal clinically important difference (MCID) values from the literature. Power and sample size data, including relevant assumptions for all calculations, are presented in Table 1.

We expect ≥ 80% power for reoperation rates, complication rates, severe complication rates, *Quick*DASH, SMFA, patient satisfaction, pain in motion and at rest, and ROM. However, we do not expect to achieve 80% power for analysis of grip strength, postoperative antibiotics use, secondary dislocation or nonunion rate, sick-leave time, fluoroscopy time, fluoroscopy DAP, or operation time (Table 1).

The minimal detectable differences (MDD) for various outcomes and study sizes are presented in Table 1. For some of the secondary and tertiary outcome measures, the MDD is smaller than the MCID meaning that the study is under-powered for proving non-inferiority between the groups. Early termination of the trial resulting in a smaller sample size would further reduce MDD, hampering the power of the study.

The final study groups consist of all participants that complete the follow-up and participants that withdraw but permit analysis; see the section “ Post-inclusion withdrawals and exclusions”. The study groups will be compared using the chi-squared test for categorical variables (reoperation, complication) and Student’s *t*-test for continuous variables.

Comparisons between the study groups will primarily be performed by “intention to treat” analyses. If large cross-over between study groups occurs, “as treated” analyses will be performed as well. Subgroup analysis stratified by Mayo classification, age, gender, open fracture, and high energy trauma is planned for all outcomes.

### Ethics

The study was approved by the Swedish Ethical Review Authority (DNR: 2021–06822-01). The associated risks are primarily post- or peri-operative complications. As both interventions are standard care at our department, no additional such risk is implied by the trial. One additional risk is the potentially harmful effect of radiation received from the radiograph at 6 months, as this is not part of standard care. The additional effective radiation dose is estimated to 0.001 mSv and minimized by only using one projection. The more rigorous follow-up could potentially enhance rehabilitation and health for included participant compared to standard care. Future patients may benefit from improved future treatment recommendations based on study results. Using data from a previous study [16], the number needed to harm is estimated to 6.8 for reoperations and 3.1 for complications when TBW is chosen over CF. We believe that the potential benefits for study participants and future patients outweigh the additional risks for the participants in the study.

### Oversight and monitoring

The principal investigator DW and co-authors GC, IK, and CR coordinate the study at all study centres and meet monthly. The day-to-day management of the study will be performed by the steering committee with support from administrative staff from the standard healthcare system. The surgical methods in this trial have been used extensively at our clinic and are considered low-risk; thus, there is no need for a data monitoring committee. Neither the steering committee, authors, nor the sponsor has any competing interests. No external endpoint adjudication system will be used.

### Data handling

All data will be collected using the participants’ unique national identification numbers, then entered into REDCap by the assessor, and stored securely on servers provided by Lund University accessible only by the steering committee. Before interim analysis, and at the end of the trial, the steering committee will transfer collected data to a pseudonymous database using the unique participant ID provided at randomization to discriminate data. This database will be stored electronically behind firewalls provided by the sponsor. The only registry of the key to the participant ID and corresponding national identification number will be stored physically in a safe only accessible by the principal investigator.

## Discussion

Migration of K-wires may lead to reoperations following TBW fixation of olecranon fractures [48]. Using CF without K-wires eliminates the risk of migrating K-wires and retrospective studies have favoured this method [7, 16, 49]. Phadnis et al. advocate a similar method using non-metal sutures [50, 51], although reserving this technique for Mayo type 2a fractures. They reported a reoperation rate after suture fixation in 1 of 41 consecutive participants, though mixing fractures and osteotomies [8]. The same group runs an RCT which is currently recruiting participants [52]. A biomechanical cadaver study showed similar fixation strength following CF and TBW, theoretically rendering K-wires superfluous [53]. Based on these findings in retrospective and experimental settings, we designed the current RCT to provide level 1 evidence examining outcomes following CF vs. TBW. We have chosen not to include plate fixation as a comparator, as that method is not used for Mayo type 2a and 2b fractures at our clinic. Thus, including plate fixation would make the implementation of this study in our healthcare setting ethically difficult.

The planned study size is sufficient for detecting a clinically significant difference in the primary outcome, reoperation rates, if that difference is as big as in a retrospective study from our department [7]. We expect a high availability of participants for analysis. We do not expect missing data on reoperations due to participants moving out of the catchment area to differ from the previous retrospective study, on which the sample size calculation is based [16]. The retrospective study that underlies sample size calculations only included elderly subjects, which differs from the planned study. We believe that the reoperation rate in the planned RCT may be higher than in the retrospective study, as younger participants with high demands are more likely to require reoperations. However, it is also possible that K-wire migration will be less likely to occur in participants with better bone quality. Higher reoperation rates than assumed would yield higher statistical power to demonstrate a difference in reoperation rates.

The trial could be terminated early due to large differences in reoperations, complications, or *Quick*DASH scores between study groups, which would negatively affect statistical power for analyses of other outcomes. Early termination of the study will hamper its strength to draw conclusions on these outcomes, but we do not think it would be ethically justifiable to continue recruitment if the postulated exclusion criteria are met.

We believe this trial to be an ethically sound way of providing level 1 evidence comparing CF with TBW in Mayo type 2a and 2b fractures. It has the potential to influence the choice of treatment for these patients, thus improving patient outcomes and health.

## Trial status, timeline, and protocol amendments

The current protocol is the first version (1.1) of the protocol. Recruitment of participants began in January 2023. Based on statistics from previous years, we expect to recruit 50 subjects per year and thus recruitment is expected to end in 2027. The end of follow-up of the last recruited participant is expected to occur in 2030. No amendments have been made. In the case of future amendments to the protocol, the Swedish Ethical Review Authority will be informed and the ClinicalTrials.gov registry will be updated. Journals where study results or the protocol are published will be contacted, and a list of amendments will be included in future versions. Participant will be informed if affected by the amendment.

### Supplementary Information

**Additional file 1. **

**Additional file 2. **

**Additional file 3. **SPIRIT Checklist for *Trials.*

## Data Availability

This protocol can be accessed through open access by any party. Only the steering committee will have access to the final trial dataset. Anonymous group level data may be shared for non-commercial uses, if ethically sound, upon request to the authors.

## Abbreviations

WOW-OK: The With Or Without Olecranon K-wire
SPIRIT: Standard Protocol Items: Recommendations for Interventional Trials
TBW: Tension band wiring
CF: Cerclage fixation
*Quick*DASH: Shortened version of The Disabilities of Arm, Shoulder and Hand score
ISRCTN: International Standard Randomised Controlled Trial Number
RCT: Randomized controlled trial
SMFA: Short Musculoskeletal Function Assessment
bSMFA: Bother index in the SMFA
dSMFA: Disability index in the SMFA
VAS: Visual analogue scale
HAKIR: Swedish national quality register for hand surgery
ROM: Range of motion
DAP: Dose area product
MCID: Minimal clinically important difference
SD: Standard deviation
MDD: Minimal detectable difference
ICMJE: International Committee of Medical Journal Editors

## References

[1] Duckworth AD, Clement ND, Aitken SA, Court-Brown CM, McQueen MM (2012). The epidemiology of fractures of the proximal ulna. Injury.

[2] Morrey BF (1995). Current concepts in the treatment of fractures of the radial head, the olecranon, and the coronoid. Instr Course Lect.

[3] Schatzker J, Schatzker J, Tile M (2005). Fractures of the Olecranon (12–B1). The Rationale of Operative Fracture Care.

[4] Tornetta P, Ricci WM, Ostrum RF, McQueen MM, McKee MD, Court-Brown CM (2020). Rockwood and Green's fractures in adults.

[5] Chalidis BE, Sachinis NC, Samoladas EP, Dimitriou CG, Pournaras JD (2008). Is tension band wiring technique the “gold standard” for the treatment of olecranon fractures? A long term functional outcome study. J Orthop Surg Res.

[6] Claessen FM, Braun Y, Peters RM, Dyer G, Doornberg JN, Ring D (2016). Factors associated with reoperation after fixation of displaced olecranon fractures. Clin Orthop Relat Res.

[7] Karlsson MK, Hasserius R, Besjakov J, Karlsson C, Josefsson PO (2002). Comparison of tension-band and figure-of-eight wiring techniques for treatment of olecranon fractures. J Shoulder Elbow Surg.

[8] Phadnis JS, Vaughan A, Luokkala T, Peters J, Watson JJ, Watts A (2020). Comparison of all suture fixation with tension band wiring and plate fixation of the olecranon. Shoulder Elbow.

[9] Romero JM, Miran A, Jensen CH (2000). Complications and reoperation rate after tension-band wiring of olecranon fractures. J Orthop Sci.

[10] Duckworth AD, Clement ND, White TO, Court-Brown CM, McQueen MM (2017). Plate versus tension-band wire fixation for olecranon fractures: a prospective randomized trial. J Bone Joint Surg Am.

[11] Matar HE, Ali AA, Buckley S, Garlick NI, Atkinson HD (2014). Surgical interventions for treating fractures of the olecranon in adults. Cochrane Database Syst Rev..

[12] Duckworth AD, Bugler KE, Clement ND, Court-Brown CM, McQueen MM (2014). Nonoperative management of displaced olecranon fractures in low-demand elderly patients. J Bone Joint Surg Am.

[13] Gallucci GL, Piuzzi NS, Slullitel PA, Boretto JG, Alfie VA, Donndorff A (2014). Non-surgical functional treatment for displaced olecranon fractures in the elderly. Bone Joint J..

[14] Parker MJ, Richmond PW, Andrew TA, Bewes PC (1990). A review of displaced olecranon fractures treated conservatively. J R Coll Surg Edinb.

[15] Veras Del Monte L, Sirera Vercher M, Busquets Net R, Castellanos Robles J, Carrera Calderer L, Mir BX (1999). Conservative treatment of displaced fractures of the olecranon in the elderly. Injury.

[16] Wenger D, Cornefjord G, Rogmark C. Cerclage fixation without K-wires is associated with fewer complications and reoperations compared with tension band wiring in stable displaced olecranon fractures in elderly patients. Arch Orthop Trauma Surg. 2021. 10.1007/s00402-021-04027-3. 10.1007/s00402-021-04027-3 PMC9474339 34236459

[17] Duckworth AD. A trial of plate fixation versus tension band wire for olecranon fractures. 2010. https://ClinicalTrials.gov/show/NCT01391936.

[18] Midtgaard KS. Operative Treatment of Olecranon Fractures. 2017. https://ClinicalTrials.gov/show/NCT03280602.

[19] Duckworth AD. A trial of non-operative versus operative management of olecranon fractures in the elderly. 2010. https://ClinicalTrials.gov/show/NCT01397643. 10.1302/0301-620X.99B7.BJJ-2016-1112.R2 28663405

[20] Frihagen F. Treatment of Olecranon Fractures in the Elderly. 2021. https://ClinicalTrials.gov/show/NCT04670900.

[21] Hospital TU, Hospital HUC, Hospital TU, Hospital OU, Hospital KU, Hospital SC, et al. Scandinavian Olecranon Research in the Elderly. 2020. https://ClinicalTrials.gov/show/NCT04401462.

[22] Qvist A. Suture fixation versus tension band wiring of simple displaced olecranon fractures. 2019. https://ClinicalTrials.gov/show/NCT04189185. 10.61409/A01240038 39575943

[23] Cook J, Watts A (2020). A comparison of two methods of surgical fixation for the treatment of simple olecranon fractures in adults. ISRCTN registry.

[24] Camino Willhuber G, Slullitel P, Taype Zamboni D, Albergo J, Terrasa S, Piuzzi N (2020). Validation of a modified Clavien-Dindo Classification for postoperative complications in orthopedic surgery. Rev Fac Cien Med Univ Nac Cordoba.

[25] Dindo D, Demartines N, Clavien PA (2004). Classification of surgical complications: a new proposal with evaluation in a cohort of 6336 patients and results of a survey. Ann Surg.

[26] Hudak PL, Amadio PC, Bombardier C (1996). Development of an upper extremity outcome measure: the DASH (disabilities of the arm, shoulder and hand) [corrected]. The Upper Extremity Collaborative Group (UECG). Am J Ind Med..

[27] Ponzer S, Skoog A, Bergstrom G (2003). The Short Musculoskeletal Function Assessment Questionnaire (SMFA): cross-cultural adaptation, validity, reliability and responsiveness of the Swedish SMFA (SMFA-Swe). Acta Orthop Scand.

[28] Gummesson C, Ward MM, Atroshi I (2006). The shortened disabilities of the arm, shoulder and hand questionnaire (QuickDASH): validity and reliability based on responses within the full-length DASH. BMC Musculoskelet Disord.

[29] Williams N (2016). The Short Musculoskeletal Function Assessment (SMFA) questionnaire. Occup Med (Lond).

[30] (HAKIR) Nqrfhs. National manual for measuring motion and strength in the elbow, forearm and hand 2019. Available from: https://hakir.se/wp-content/uploads/2019/03/Manual-for-rorelse-styrka-Version-1-2016_Eng.pdf.

[31] Powell AJ, Farhan-Alanie OM, McGraw IWW (2019). Tension band wiring versus locking plate fixation for simple, two-part Mayo 2A olecranon fractures: a comparison of post-operative outcomes, complications, reoperations and economics. Musculoskelet Surg.

[32] Franchignoni F, Vercelli S, Giordano A, Sartorio F, Bravini E, Ferriero G (2014). Minimal clinically important difference of the disabilities of the arm, shoulder and hand outcome measure (DASH) and its shortened version (QuickDASH). J Orthop Sports Phys Ther.

[33] Iordens GIT, Den Hartog D, Tuinebreijer WE, Eygendaal D, Schep NWL, Verhofstad MHJ (2017). Minimal important change and other measurement properties of the Oxford Elbow Score and the Quick Disabilities of the Arm, Shoulder, and Hand in patients with a simple elbow dislocation; validation study alongside the multicenter FuncSiE trial. PLoS ONE.

[34] Mintken PE, Glynn P, Cleland JA (2009). Psychometric properties of the shortened disabilities of the Arm, Shoulder, and Hand Questionnaire (QuickDASH) and Numeric Pain Rating Scale in patients with shoulder pain. J Shoulder Elbow Surg.

[35] Polson K, Reid D, McNair PJ, Larmer P (2010). Responsiveness, minimal importance difference and minimal detectable change scores of the shortened disability arm shoulder hand (QuickDASH) questionnaire. Man Ther.

[36] Sorensen AA, Howard D, Tan WH, Ketchersid J, Calfee RP (2013). Minimal clinically important differences of 3 patient-rated outcomes instruments. J Hand Surg Am.

[37] Ekholm R, Tidermark J, Tornkvist H, Adami J, Ponzer S (2006). Outcome after closed functional treatment of humeral shaft fractures. J Orthop Trauma.

[38] Norman GR, Sloan JA, Wyrwich KW (2003). Interpretation of changes in health-related quality of life: the remarkable universality of half a standard deviation. Med Care.

[39] Zwerus EL, Willigenburg NW, Scholtes VA, Somford MP, Eygendaal D, van den Bekerom MP (2019). Normative values and affecting factors for the elbow range of motion. Shoulder Elbow.

[40] Lustenberger T, Leonardy R, Marzi I, Frank J (2020). Outcome after surgical treatment of complex elbow fractures: a single-center follow-up study. Eur J Trauma Emerg Surg.

[41] Bohannon RW (2019). Minimal clinically important difference for grip strength: a systematic review. J Phys Ther Sci.

[42] Papagelopoulos PJ, Morrey BF (1994). Treatment of nonunion of olecranon fractures. J Bone Joint Surg Br.

[43] Prevention NCIsDoC. National Cancer institute’s Clinical Trial Randomization Tool. Available from: https://ctrandomization.cancer.gov/home/.

[44] Harris PA, Taylor R, Minor BL, Elliott V, Fernandez M, O'Neal L (2019). The REDCap consortium: Building an international community of software platform partners. J Biomed Inform.

[45] Harris PA, Taylor R, Thielke R, Payne J, Gonzalez N, Conde JG (2009). Research electronic data capture (REDCap)–a metadata-driven methodology and workflow process for providing translational research informatics support. J Biomed Inform.

[46] Kloen P,Ring D. Tension band wiring, 2018. Available from: https://surgeryreference.aofoundation.org/orthopedic-trauma/adult-trauma/proximal-forearm/ulna-articular-olecranon/tension-band-wiring.

[47] Chow SC, Shao J, Wang H, Lokhnygina Y. Sample size calculations in clinical research. New York: Taylor & Francis; 2017.

[48] Saeed ZM, Trickett RW, Yewlett AD, Matthews TJ (2014). Factors influencing K-wire migration in tension-band wiring of olecranon fractures. J Shoulder Elbow Surg.

[49] Roukoz S, Bayoud W (2016). WIRELESS TENSION BAND WIRING FOR OLECRANON FRACTURES. Case Series J Med Liban.

[50] Phadnis J, Eves T, Watts AC. Tension suture fixation of olecranon fractures. JBJS Essent Surg Tech. 2021;11(2). 10.2106/JBJS.ST.20.00042. 10.2106/JBJS.ST.20.00042 PMC8280026 34277131

[51] Phadnis J, Watts AC (2017). Tension band suture fixation for olecranon fractures. Shoulder Elbow.

[52] Cook E, James S, Watts AC (2023). The St. A randomized controlled trial to compare clinical and cost-effectiveness of suture fixation versus tension band wiring for simple olecranon fracture fixation in adults: The Simple Olecranon Fracture Fixation Trial (SOFFT) protocol. Bone Jt Open..

[53] Murphy DF, Greene WB, Gilbert JA, Dameron TB (1987). Displaced olecranon fractures in adults. Biomechanical analysis of fixation methods. Clin Orthop Relat Res..

