# Supplementary material for: The With Or Without Olecranon K-wire (WOW OK) Trial of tension band wire fixation versus cerclage fixation without K-wires in displaced stable olecranon fractures: study protocol for a randomized controlled trial
Source: Trials. 2023 Aug 29;24:559. doi: 10.1186/s13063-023-07566-9 (PMC10464474; doi:10.1186/s13063-023-07566-9)
Supplement: Supplementary file 1 — Additional file 1. [file 13063_2023_7566_MOESM1_ESM.pdf]

SAMTYCKE TILL DELTAGANDE I STUDIEN OCH MEDGIVANDE TILL JOURNAL- OCH DATA- HANTERING

Jag har informerats muntligt om studien och jag har läst den skriftliga informationen. Jag har fått svar på mina frågor och jag samtycker till att deltaga i studien. Mitt deltagande är frivilligt och jag vet att jag kan avbryta när som helst utan förklaring, utan att det påverkar mitt omhändertagande.
Jag har fått information om att de uppgifter som samlats in om mig i studien kommer att behandlas konfidentiellt, på ett sådant sätt att min identitet inte kommer att avslöjas för obehöriga. Jag tillåter att i studien insamlade uppgifter i original kan utlämnas av ansvarig läkare till medicinska kontrollmyndigheter, Etikprövningsmyndigheten eller till personal som arbetar med studien, allt under förutsättning att sedvanlig sekretess upprätthålls.
Jag är informerad om rättigheten att få registerutdrag en gång per år över vilka personuppgifter som är insamlade. Jag har dessutom informerats om rättigheten att få felaktiga uppgifter rättade eller borttagna.

………………………………… …………………………………
Ort/datum (ifylles av patient) Patientens underskrift

…………………………………
Namnförtydligande (patient)

………………………………… …………………………………
Ort/datum (ifylles av informerande person) Informerande persons underskrift

…………………………………
Namnförtydligande (informerande person)

SKRIFTLIGT SAMTYCKE FÖR TILLGÅNG TILL PATIENTJOURNAL

Jag har informerats om och samtycker härmed till att oberoende personal som kontrollerar studien och eventuell svensk eller utländsk kontrollmyndighet får jämföra uppgifter i studien med uppgifter som finns i min patientjournal. Detta får ske under förutsättning att den information som därvid blir tillgänglig inte förs vidare och att endast de uppgifter som har betydelse för studien kommer att kontrolleras.

…………………………………… …………………………………
Datum (ifylles av patient) Patientens underskrift
