# Supplementary material for: The With Or Without Olecranon K-wire (WOW OK) Trial of tension band wire fixation versus cerclage fixation without K-wires in displaced stable olecranon fractures: study protocol for a randomized controlled trial
Source: Trials. 2023 Aug 29;24:559. doi: 10.1186/s13063-023-07566-9 (PMC10464474; doi:10.1186/s13063-023-07566-9)
Supplement: Supplementary file 2 — Additional file 2. [file 13063_2023_7566_MOESM2_ESM.pdf]

Utfall efter operation av fraktur på armbågsspetsen: en randomiserad prospektiv jämförelse mellan fixation med cerklage och tension band wire-teknik

PATIENTINFORMATION

Förfrågan om deltagande i en vetenskaplig studie

Vi vill fråga Dig som har råkat råkat ut för en armbågsfraktur som behöver opereras om Du vill delta i en vetenskaplig studie. Titeln på denna studie är "Utfall efter operation av fraktur på armbågsspetsen: en randomiserad prospektiv jämförelse mellan fixation med cerklage och tension band wire-teknik". Forskningshuvudman för studien är Region Skåne. Med forskningshuvudman menas den organisation som är ansvarig för projektet. Studien är godkänd av Etikprövningsmyndigheten. Diarienummer för prövningen hos Etikprövningsmyndigheten är 2022-06951-02

Bakgrundsinformation om studien

Operation rekommenderas för de flesta felställda frakturer på armbågsspetsen. Det är oklart vilken operationsmetod som ger bäst resultat. Vi jämför nu två etablerade operationsmetoder: att stabilisera frakturen med ståltråd och stift eller enbart med ståltråd.

Deltagande

Alla patienter som inkommer till SUS med en fraktur på armbågsspetsen som behöver opereras och uppfyller specifika villkor (bl.a. minst 18 års ålder, fraktur som är mindre än 14 dagar gammal) tillfrågas om att delta i studien. Deltagande är helt frivilligt.

Hur går studien till?

Innan operationen bestäms metoden för stabilisering av frakturen slumpmässigt (lottning). Efter operationen får du ett gips över armbågen i 2 veckor och erbjuds rehabiliterings-träning. Du kommer att få hjälp av fysioterapeut att träna upp rörlighet och styrka. Det är samma efterbehandling efter båda metoderna.
Efter din skada kommer du att få återbesök efter 2 veckor, 6 veckor, 3 månader, 1 år och 3 år. Läkningsförloppet följs med röntgen samt undersökningar av rörlighet och greppstyrka.
Dessutom kommer vi att be dig att fylla i frågeformulär om den skadade armbågens funktion. Vi vill även undersöka hur länge du eventuellt behöver vara sjukskriven från arbetet. Viss information hämtar vi från din journal, t.ex. tekniska detaljer om din operation.
Vi är väl förtrogna med båda operationsmetoderna, och de är standardbehandling vid denna typ av fraktur. Att vara med i studien innebär alltså att du kommer att genomgå ”vanlig” behandling. Skillnaden är att en av de två operationsmetoderna väljs slumpmässigt, istället för att operatören väljer metod.
Den normala uppföljningen är ett läkarbesök efter 2-3 veckor och därefter rehabilitering med fysioterapeut. Uppföljningen om du deltar i studien pågår under längre tid än den normala och din armbåge undersöks också en gång med röntgen, vilket inte görs rutinmässigt för personer som inte deltar.

Risker och eventuella biverkningar

Frakturer och operationer är som regel smärtsamma i början. Oavsett operationsmetod finns risk för infektion eller tryckskada på skinnet från gipset. Det finns även en risk att frakturen inte läker, vilken dock är betydligt större om man inte opererar. Implantatet kan även bli störande under skinnet när svullnaden minskar efter läkningen. Vi är vana vid att operera med båda metoderna på ortopedkliniken i Malmö-Lund. Vi vill jämföra metoderna, och se om någon av dem leder till mindre komplikationer än den andra. De som deltar i studien gör en extra röntgen av armbågen efter 6 månader. Stråldosen från den extra röntgen är så låg att den inte bedöms leda till någon hälsorisk. Studien är granskad och godkänd av Etikprövningsmyndigheten.

Finns det fördelar med att delta?

Deltagare i studien får ingen särskild ersättning. Studien innebär dock en mer noggrann uppföljning efter operationen än det normala. Genom att delta i studien bidrar man till ökad kunskap som i slutändan kan leda till förbättring av vården för andra som råkar ut för samma skada.

Sekretess och databearbetning

All information som samlas in datorregistreras. Region Skåne är personuppgifts-ansvarig för detta register. Om du samtycker till att delta samtycker du också till att personuppgiftsbehandling sker. De uppgifter som registreras är personnummer, namn, röntgenbilder, och uppgifter kring din hälsa från fysioterapeutens uppföljning , projektets frågeformulär och din journal. Ditt deltagande i studien skyddas av sjukvårdens sekretess. När uppgifterna samlats in kommer all fortsatt bearbetning av data ske avidentifierad och inga resultat kommer att kunna härledas till dig själv. Berörda myndigheter har rätt att granska insamlade data enligt gällande lagar och föreskrifter.

Dina journalhandlingar förvaras av Region Skåne i din patientjournal. Uppgifter från frågeformulären och uppföljningen samlas in ett datorsystem som skyddas av Lunds Universitet. Datan kommer sedan överföras till en databas som förvaras i ett kodlåst brandskyddsskåp av den ansvariga läkaren (Daniel Wenger, se nedan) och raderas ur Lunds Universitets datorsystem. De samlade uppgifterna från journalen, frågeformulär och undersökningar förvaras på en kodlåst datafil där dina personuppgifter är borttagna och ersatta med ett löpnummer. En nyckel för koppling av personuppgiter och löpnummer förvaras på en annan kodlåst datafil. De två datafilerna förvaras åtskilda från varandra av den ansvarige läkaren (Daniel Wenger, se nedan). Efter 10 år förstörs frågeformulären och datafilen arkiveras via Lunds Universitet i enlighet med GDPR-lagen (General Data Protection Regulation). Om du vill ta del av dina individuella uppgifter ska du kontakta den ansvarige forskaren (Daniel Wenger, se nedan).

Personuppgifter hanteras i enlighet med Dataskyddsförordningen (2016/679) som också är känd som GDPR-lagen (General Data Protection Regulation). Enligt GDPR har du rätt att kostnadsfritt få ta del av de uppgifter om Dig som hanteras i projektet, och vid behov få eventuella fel rättade. Du kan också begära att uppgifter om dig raderas samt att behandlingen av dina personuppgifter begränsas. Rätten till radering och tillbegränsning av behandling av personuppgifter gäller dock inte när uppgifterna är nödvändiga för den

aktuella forskningen. Du har rätt att ansöka om information från personuppgiftsbehandlingen genom att skriva till **Dataskyddsombudet, Region Skåne, 291 89 Kristianstad**. Din ansökan måste vara undertecknad av dig själv. Du har också rätt att få eventuella felaktiga personuppgifter rättade eller ta bort dina uppgifter om du inte vill att de står kvar i registret.

Försäkring och ersättning

Alla patienter som deltar i studien är försäkrade mot oönskade medicinska händelser i enlighet med gällande lagstiftning genom Landstingets Ömsesidiga Försäkringsbolag (LÖF). Försäkringen är densamma som du får om du inte deltar i studien och gäller om oönskade medicinska händelser uppstår som följd av vården du får.

Frivilligt deltagande

Ditt deltagande i studien är helt frivilligt.

Om du väljer att delta kan du när som helst avbryta ditt deltagande, utan att du behöver ange någon orsak. Din framtida vård påverkas inte av om du avbryter. Även din läkare kan avbryta ditt deltagande om han/hon bedömer att det äventyrar din säkerhet, eller om till exempel skulle flytta utomlands och inte kan fullfölja uppföljningen. Om du bestämmer dig för att avbryta ditt deltagande är det bara att kontakta den ansvariga läkaren (Daniel Wenger, se nedan).

Om du tackar nej till att delta kommer du att få den behandling som operatören väljer i samråd med dig. Detta påverkar inte din behandling eller bemötandet på något sätt.

Deltagare måste fylla i en samtyckesblankett.

Genom att underteckna blanketten bekräftar du att du har fått skriftlig och muntlig information om studien, och att du förstår informationen och vill delta.

Tveka inte att kontakta undertecknad om du har frågor om studien eller dina rättigheter som deltagare, om du vill få mer information eller om du skulle uppleva biverkningar.

Malmö 2023-01-01

Daniel Wenger
Överläkare
VO Ortopedi SUS
Inga Marie Nilssons gata 22
205 02 Malmö
daniel.wenger@med.lu.se
tel: 040-332668
